# Hydroxytyrosol as a Multitarget Neuroprotective Agent: Molecular Mechanisms, Pharmacokinetics and Therapeutic Potential in Neurodegenerative Diseases

**DOI:** 10.3390/molecules31173113

**Published:** 2026-09-05

**Authors:** Pura Ballester-Navarro, Ana María García-Muñoz, Desirée Victoria-Montesinos, Pilar Zafrilla

**Affiliations:** Faculty of Pharmacy and Nutrition, UCAM Universidad Católica de Murcia, 30107 Murcia, Spain; pballester@ucam.edu (P.B.-N.); amgarcia13@ucam.edu (A.M.G.-M.); mpzafrilla@ucam.edu (P.Z.)

**Keywords:** hydroxytyrosol, neurodegenerative diseases, oxidative stress, mitochondrial dysfunction, nutraceuticals

## Abstract

Neurodegenerative diseases arise from interacting oxidative, inflammatory, mitochondrial, and proteostatic disturbances. Hydroxytyrosol (HT), an olive phenol, has been proposed as a multitarget neuroprotective compound. This narrative review integrates HT chemistry, parent/metabolite pharmacokinetics, blood–brain barrier (BBB) evidence, mechanisms, disorder-specific models, and human studies. Direct HT evidence is strongest for nuclear factor erythroid 2-related factor 2/antioxidant response element (Nrf2/ARE) activation and experimental modulation of α-synuclein; support for AMP-activated protein kinase (AMPK)/sirtuin 1 (SIRT1)/peroxisome proliferator-activated receptor gamma coactivator 1-alpha (PGC-1α), mitochondrial protection, nuclear factor-kappa B (NF-κB)-related inflammation, and amyloid-β (Aβ) is predominantly preclinical, whereas tau, autophagic flux, and ubiquitin–proteasome effects remain preliminary. Oral HT is rapidly absorbed but extensively conjugated, and no study has quantified parent HT or its major metabolites in the human brain or cerebrospinal fluid after oral supplementation. Isolated-HT trials show systemic antioxidant or anti-inflammatory biomarker effects, while cognitive findings derive mainly from phenolic-rich olive matrices and cannot be assigned to HT alone. No disease-modifying efficacy has been established for isolated HT in Alzheimer’s disease (AD), Parkinson’s disease (PD), or related disorders. HT is therefore a mechanistically plausible candidate, but human brain exposure, dose–response, and efficacy require adequately powered disease-specific trials.

## 1. Introduction

Neurodegenerative diseases are a major and increasing cause of disability and death. AD, PD, amyotrophic lateral sclerosis, Huntington’s disease, and related dementias cause progressive neuronal loss, cognitive or motor impairment, and substantial societal burden [1,2,3]. Their pathogenesis is multifactorial: oxidative stress, mitochondrial dysfunction, neuroinflammation, impaired proteostasis, excitotoxicity, and abnormal protein aggregation interact rather than acting independently [4,5,6]. This network biology motivates multitarget strategies, while also requiring that each proposed target be graded according to the supporting evidence.

HT (3,4-dihydroxyphenylethanol) is an olive phenol associated with Mediterranean dietary patterns. Experimental studies report redox, inflammatory, mitochondrial, apoptotic, and protein-aggregation effects [6,7,8,9]. However, oral exposure is dominated by conjugated metabolites, and central nervous system access is supported mainly by animal and in vitro barrier models rather than human brain measurements [8,10,11].

Clinical translation is limited by extensive first-pass metabolism, matrix- and dose-dependent exposure, and substantial interindividual variability [10]. Most neurodegeneration evidence remains cellular or animal; human studies largely assess pharmacokinetics or systemic biomarkers, not disease progression [12].

Prior reviews have emphasized broad antioxidant/anti-inflammatory actions or a single disorder [8,13]. The present review adds four elements: integration of parent HT with circulating metabolites and BBB evidence; an explicit hierarchy of mechanism-specific support; separation of isolated HT, HT-enriched oil, and mixed matrices for causal attribution; and clinically actionable dose, biomarker, and trial-design gaps.

Accordingly, this review evaluates the chemical and pharmacokinetic basis of HT, ranks its proposed neuroprotective mechanisms, compares AD and PD with more preliminary disorders, and assesses whether current human evidence supports clinical translation.

### Review Methodology

This narrative review searched PubMed/MEDLINE and Crossref from database inception to 23 July 2026, supplemented by backward reference screening and a ClinicalTrials.gov search. Search combinations joined ‘hydroxytyrosol’ with neurodegeneration, Alzheimer, Parkinson, amyotrophic lateral sclerosis, Huntington, cognition, pharmacokinetics, metabolites, blood–brain barrier, Nrf2, NF-κB, NLRP3, mitochondria, aggregation, autophagy, monoamine oxidase, and clinical trial. English-language peer-reviewed primary studies were prioritized; reviews were used for context. Evidence was classified as direct HT-specific, HT-rich/mixed-matrix, or indirect pathway evidence, with human data considered separately from cellular and animal findings. Because this was a narrative rather than systematic review, no formal risk-of-bias tool or quantitative meta-analysis was applied.

## 2. Chemical and Pharmacokinetic Profile of Hydroxytyrosol

### 2.1. Chemical Structure and Redox Properties

#### 2.1.1. Catecholic Structure

HT is structurally defined by the presence of a catechol moiety, consisting of two hydroxyl groups in the ortho position on the aromatic ring, which represents the principal determinant of its redox behavior and antioxidant potential. This ortho-dihydroxyl configuration enhances electron density within the aromatic system and facilitates rapid interaction with ROS [14,15], thereby conferring high radical scavenging efficiency and redox reactivity [16,17].

Moreover, the catechol structure enables HT to participate in multiple biochemical interactions beyond simple redox reactions, including binding to protein aggregates and modulation of amyloidogenic pathways [18,19], which further highlights the functional relevance of this structural motif in biological systems [20]. Collectively, these features position HT among the most reactive naturally occurring phenolic antioxidants present in olive-derived matrices [21].

#### 2.1.2. Hydrogen-Donating Capacity

The antioxidant activity of HT is largely mediated by its hydrogen atom donation capacity, which allows neutralization of free radicals and termination of oxidative chain reactions [22,23]. The presence of two hydroxyl groups in the catechol ring significantly enhances this capacity compared to monophenolic analogues, enabling more efficient scavenging of ROS and reactive nitrogen species [24,25].

Importantly, HT is capable of sequential hydrogen donation, whereby each hydroxyl group can independently contribute to radical neutralization, thereby increasing its overall antioxidant efficiency and prolonging its protective effects under oxidative stress conditions [26,27]. This dual hydrogen-donating mechanism is considered a key factor underlying its superior activity relative to structurally simpler phenols such as tyrosol [28].

#### 2.1.3. Resonance and Radical Stability

Following hydrogen atom transfer, HT forms a phenoxyl radical that is stabilized through extensive resonance delocalization, allowing redistribution of the unpaired electron across the aromatic ring and significantly reducing its reactivity [29]. This resonance stabilization is a defining feature of catechol-containing phenols and is directly associated with their ability to prevent propagation of oxidative chain reactions [15,30].

Additionally, intramolecular hydrogen bonding between the hydroxyl groups further stabilizes the radical intermediate, enhancing its persistence and limiting secondary radical formation [31]. The combined effects of resonance and hydrogen bonding explain the thermodynamic stability of HT-derived radicals and its effectiveness as a chain-breaking antioxidant [32,33].

#### 2.1.4. Comparison with Other Olive Oil Phenols

The antioxidant capacity of HT has been extensively compared with other olive oil phenolics, including tyrosol and p-coumaric acid, which lack the catechol structure and therefore exhibit reduced hydrogen-donating ability and weaker radical stabilization [34,35]. As a consequence, HT generally displays superior radical scavenging activity and redox efficiency under a wide range of experimental conditions [36].

However, it is important to note that phenolic mixtures can exhibit synergistic or additive effects, with certain combinations of monophenols demonstrating comparable or even enhanced antioxidant activity relative to HT alone [36,37], depending on the system evaluated. These findings indicate that antioxidant behavior in olive oil is not solely dependent on individual compounds but rather on complex molecular interactions within the phenolic matrix [38].

### 2.2. Absorption, Metabolism and BBB Permeability

HT is rapidly absorbed after oral intake but also undergoes extensive early biotransformation. As a result, systemic circulation is dominated by conjugated metabolites rather than the free molecule. This feature is central to both its pharmacokinetic profile and the interpretation of its potential biological effects at peripheral and cerebral levels [39].

#### 2.2.1. Oral Bioavailability

Available evidence in humans indicates that HT is absorbed rapidly after ingestion, although its bioavailability depends not only on the administered dose, but also on the food matrix in which it is incorporated. In a human trial, administration of 5 mg of HT in different matrices showed that extra virgin olive oil provided greater early plasma exposure, with a maximum plasma concentration of 3.79 ng/mL at 30 min, supporting that the lipid fraction may favor the release, stability and/or intestinal absorption of the compound [39].

Similarly, another crossover study in healthy volunteers using aqueous olive-derived supplements and enriched olive oil observed that peak plasma concentrations of the main metabolites also occurred at 30 min, and exposure increased with the administered dose. In that study, aqueous supplements allowed detection of substantial urinary metabolite recovery, although the overall profile still showed marked interindividual variability [40,41].

However, describing HT as a compound with “low bioavailability” may be misleading. The main issue is not poor absorption, but the fact that free HT is highly transient because it is rapidly converted into conjugated metabolites [42]. Accordingly, low plasma levels of the native molecule should not be interpreted as evidence of limited absorption [39,40]. Indeed, the use of more sensitive analytical methods has enabled the detection of free HT in human plasma after extra virgin olive oil intake, whereas with conventional handling and less sensitive approaches it often appears nearly undetectable [10,43,44].

Another relevant factor influencing HT bioavailability is its endogenous formation from other olive phenolics, particularly oleuropein and related secoiridoids, which may be hydrolysed during digestion and may also be influenced by gut microbiota activity [40]. This process may contribute to the systemic availability of HT beyond the amount directly ingested as free HT. In addition, interindividual variability in gut microbiota composition has been proposed as a factor influencing HT absorption efficiency and the resulting circulating metabolite profile [10,45]. 

Therefore, from a pharmacokinetic perspective, HT should be considered a compound with rapid absorption but extensive presystemic and systemic metabolism, so that systemic exposure is not adequately represented solely by concentrations of the native molecule [8,45].

#### 2.2.2. Metabolism: Glucuronides, Sulfates and Other Derivatives

Following intestinal absorption, HT undergoes extensive phase II metabolism in enterocytes and liver, mainly through sulfation and glucuronidation. In addition, methylated and oxidised derivatives related to catechol metabolism are formed, including homovanillic acid (HVA), homovanillyl alcohol and 3,4-dihydroxyphenylacetic acid (DOPAC) [10,40].

In humans, recent studies consistently show that the predominant metabolites in plasma and urine are HT-3-O-sulfate, HT glucuronide conjugates, as well as HVA and DOPAC. For example, Bender et al. identified HVA, HT-3-O-sulfate, and DOPAC among the main metabolites detected in both plasma and urine, supporting the view that HT exposure is largely represented by metabolite profiles rather than by the parent compound alone [10,40,46]. In the study by González-Santiago et al., urinary balance showed metabolites present as free forms (44%), glucuronides (34.4%) and sulfates (21.2%), confirming that conjugation dominates the overall metabolic profile [47]. Furthermore, a recent 2025 review highlights that in systemic circulation, HT occurs mainly as conjugated metabolites and that sulfation appears to be the predominant metabolic pathway, while the free fraction represents only a minor proportion of the total circulating forms [10].

#### 2.2.3. Half-Life and Tissue Distribution

One of the most important aspects when describing this section accurately is distinguishing between the half-life of free HT and the persistence of the total metabolite pool following oral administration. Early studies using radiolabeled HT in rats after intravenous administration reported extremely rapid plasma disappearance of the native molecule, with a half-life of approximately 1–2 min, reflecting rapid metabolism and tissue uptake [48]. However, under conditions closer to dietary exposure, the picture is different. Human studies show peak plasma concentrations between 13 and 30 min, while metabolites may remain detectable for several hours, with significant urinary recovery up to 12–24 h [47].

In rats fed table olives, a 2024 study described linear pharmacokinetics of HT between approximately 2.95 and 5.85 mg/kg, with a half-life of about 2.5 h for the analyzed profile and a mean residence time of around 4 h, reflecting the kinetics of HT and its immediate metabolites rather than the native compound alone [49]. Regarding tissue distribution, preclinical evidence indicates that HT and/or its metabolites rapidly reach several organs. The main accumulation sites are liver and kidney, although measurable levels have also been reported in muscle, testis and brain. In radiolabel studies, radioactivity appeared in tissues minutes after administration, and subsequent work confirmed dose-dependent accumulation in liver, kidney and brain, even at nutritionally relevant doses [50].

Beyond classical pharmacokinetic parameters, it has been suggested that the biological persistence of HT-derived metabolites may exceed the plasma half-life of the parent compound, due to tissue retention and possible enterohepatic recirculation. Consequently, although free HT disappears rapidly from plasma, the presence of circulating metabolites may prolong systemic exposure and contribute to sustained biological activity. This observation may partly explain the discrepancy between the short plasma half-life reported in early pharmacokinetic studies and the longer-lasting biological effects described in experimental models [10,51].

#### 2.2.4. Evidence of BBB Permeability

The blood–brain barrier (BBB) evidence requires careful interpretation. Animal and human in vitro models support transport of HT or some metabolites, but no study has quantified these compounds in the human brain or cerebrospinal fluid after oral supplementation [8].

Rodent studies detected brain radioactivity or low HT concentrations after systemic administration, with greater uptake when BBB integrity was disrupted [48,52,53]. Two human in vitro BBB systems reported approximately 70% transendothelial transport in one model [54] and 65–100% cumulative basolateral recovery at 24 h in another [55]. These values describe different models and endpoints and are not estimates of oral bioavailability or human brain exposure.

A nanocarrier study reported an approximately 50% relative increase in transport versus free HT in its own BBB model [56]. This formulation effect is not directly comparable with the absolute transport percentages above and remains preclinical.

Thus, central exposure is plausible but unquantified in humans. Future work should measure parent HT and major sulfate/glucuronide metabolites in plasma and cerebrospinal fluid, relate them to validated pharmacodynamic markers, and avoid inferring brain concentrations from in vitro transport percentages [8,52,53].

#### 2.2.5. Relevance of Conjugated Forms 

The classical view that only free HT is biologically active is now increasingly considered oversimplified. Current evidence suggests that biological activity may arise from a dynamic interplay between the native molecule and its conjugated metabolites rather than from the free compound alone [8].

First, conjugated forms represent the dominant circulating species. Second, some appear to contribute to systemic distribution; for example, erythrocytes have been proposed to act as temporary reservoirs of phase II metabolites, facilitating their distribution and potential local deconjugation [57]. Third, several studies suggest that certain circulating metabolites may reach the brain or exert biological activity in neuronal models at physiologically achievable concentrations [52].

Therefore, modern reviews generally avoid statements suggesting that “free HT is the only biologically relevant form”. Instead, it is more accurate to indicate that conjugated forms are essential to explain the true systemic exposure of HT, and their relevance may derive from three non-exclusive mechanisms: intrinsic biological activity, tissue transport followed by local deconjugation, or dynamic equilibrium with small fractions of free bioactive HT [10].

The principal pharmacokinetic characteristics of HT after oral intake are summarized in Table 1.

**Figure 1:**
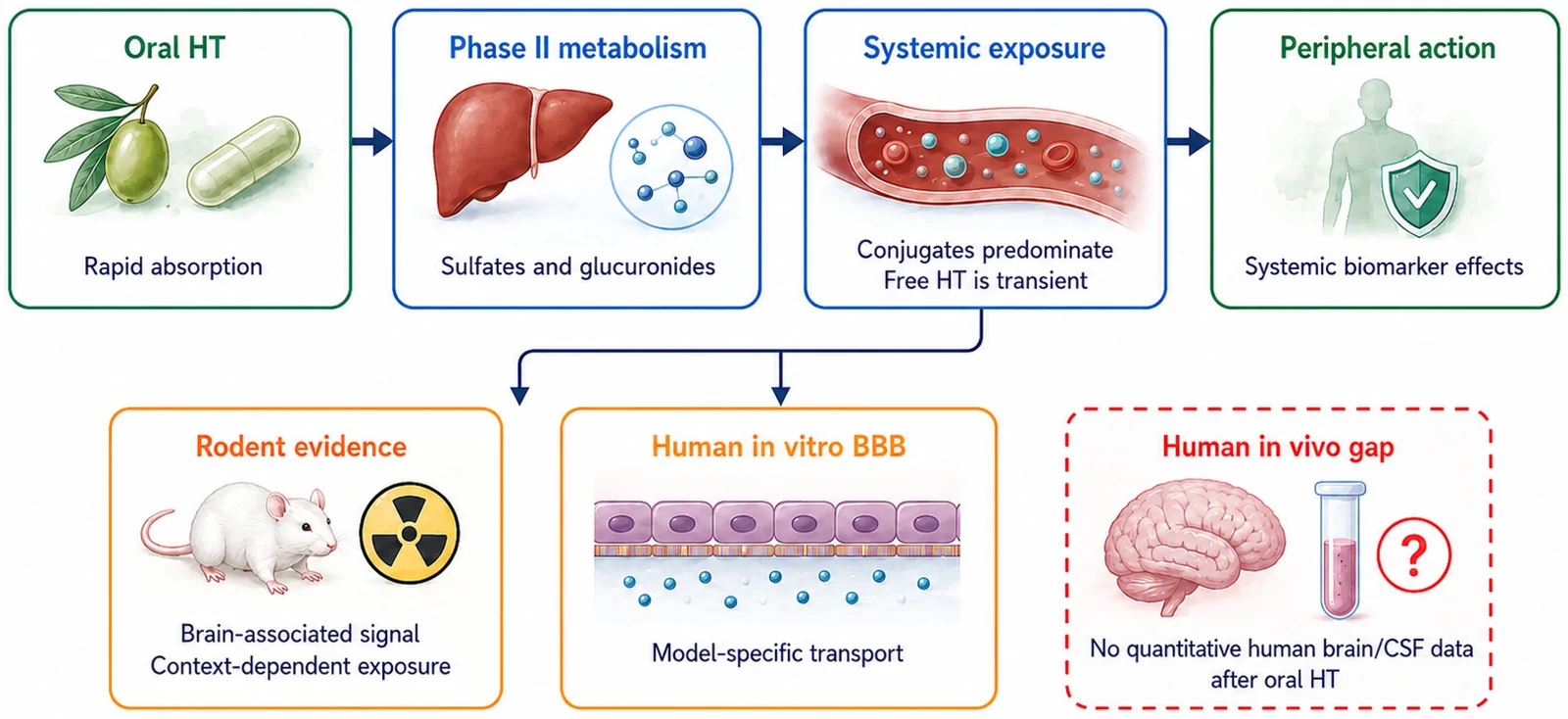
Pharmacokinetic disposition of hydroxytyrosol and hierarchy of evidence for brain exposure. Reported blood–brain barrier transport estimates derive from different experimental models and endpoints and should not be interpreted as estimates of human brain exposure. Neither parent hydroxytyrosol nor its major metabolites have been quantitatively determined in human brain or cerebrospinal fluid after oral supplementation. BBB, blood–brain barrier; CSF, cerebrospinal fluid; HT, hydroxytyrosol.

These pharmacokinetic and brain-exposure considerations are summarized in Figure 1.

## 3. Molecular Mechanisms of Neuroprotection

### 3.1. Antioxidant Signaling Pathways

HT can scavenge reactive species through its catechol structure [26,58], but this stoichiometric activity does not establish relevance at concentrations reached in neural tissue after oral intake [4,8,40,49,55,59]. Neuroprotective interpretation should therefore prioritize experimentally supported modulation of endogenous redox and mitochondrial pathways.

One of the most consistently described mechanisms is the activation of the Nrf2/ARE pathway [60,61]. Under basal conditions, Nrf2 is retained in the cytoplasm by Kelch-like ECH-associated protein 1 and targeted for proteasomal degradation. Under oxidative or electrophilic stress, Nrf2 stabilizes, translocates to the nucleus, and promotes the transcription of antioxidant and detoxifying genes [60,61]. In neuronal-like pheochromocytoma (PC12) cells, HT has been shown to increase nuclear Nrf2 levels and induce a panel of cytoprotective enzymes, including heme oxygenase-1 (HO-1), NAD(P)H quinone dehydrogenase 1, glutamate-cysteine ligase, and thioredoxin reductase. Importantly, Nrf2 silencing abolished the protective effect of HT against hydrogen peroxide- and 6-hydroxydopamine (6-OHDA)-induced injury, suggesting that Nrf2 activation is not merely associated with HT neuroprotection, but is functionally required for it [62].

This evidence is mechanistically stronger than general antioxidant-marker changes, although it remains preclinical and does not establish target engagement in the human brain [13,59,60,61,63,64].

In addition to Nrf2 activation, HT appears to modulate mitochondrial redox homeostasis. Mitochondria are both a major source and a major target of ROS in neurodegenerative diseases. Mitochondrial dysfunction amplifies oxidative damage, impairs adenosine triphosphate generation, disturbs calcium buffering, and facilitates apoptotic signaling [59,60,61]. In db/db mice, HT administration improved the expression of mitochondrial respiratory chain complexes, reduced protein oxidation in brain tissue, and increased antioxidant proteins such as HO-1, superoxide dismutase 1, and superoxide dismutase 2. These effects were accompanied by activation of the AMPK/SIRT1/PGC-1α axis, a signaling network involved in mitochondrial biogenesis, energy sensing, and oxidative stress adaptation [65,66]. Thus, HT may contribute to neuroprotection by improving mitochondrial competence rather than simply reducing oxidative markers downstream.

AMPK-dependent increases in complex IV and HO-1, reduced reactive oxygen species (ROS), and preserved viability have also been reported in high-glucose neuronal injury models [59,66,67]. Their relevance to human neurodegeneration remains to be tested.

Nicotinamide adenine dinucleotide phosphate oxidase modulation is plausible but less directly demonstrated in HT-treated neuronal models than Nrf2 activation [68,69].

Mitogen-activated protein kinase (MAPK) and phosphoinositide 3-kinase/protein kinase B (PI3K/Akt) effects vary with cell type, insult, dose, and exposure time; they should be treated as context-dependent signaling nodes [59,64,65,70].

Accordingly, support is strongest for Nrf2/ARE, moderate for AMPK/SIRT1/PGC-1α-related mitochondrial regulation, and weaker or context-dependent for NADPH oxidase, MAPK, and PI3K/Akt [64,67,69,70].

### 3.2. Anti-Inflammatory Pathways

HT-related anti-inflammatory evidence spans immune, intestinal, and neural models, but these settings are not interchangeable. The following subsections distinguish direct neurorelevant findings from peripheral or inferential support [6,29,71,72].

#### 3.2.1. Regulation of Innate Immune Signaling Pathways

The anti-inflammatory effects of HT are largely mediated through the modulation of innate immune signaling pathways that orchestrate the initiation and amplification of inflammatory responses. Among these pathways, the Toll-like receptor 4 (TLR4)/NF-κB axis appears to represent a central molecular target. Activation of TLR4 by microbial products, endogenous danger-associated molecular patterns, or tissue injury initiates a myeloid differentiation primary response protein 88-dependent signaling cascade that culminates in NF-κB activation and the transcription of numerous pro-inflammatory genes, including cytokines, chemokines, cyclooxygenase-2, and inducible nitric oxide synthase [6,73,74]. Experimental evidence consistently demonstrates that HT attenuates NF-κB activation and reduces the expression of downstream inflammatory mediators. In a dextran sulfate sodium (DSS)-induced colitis model, Tang et al. demonstrated that HT significantly inhibited TLR4 expression and NF-κB activation, resulting in reduced colonic inflammation, restoration of epithelial barrier integrity, and improvement of histopathological alterations associated with intestinal injury [73]. These findings are consistent with previous studies showing that HT suppresses NF-κB-dependent inflammatory responses and reduces the expression of pro-inflammatory mediators in activated immune cells [6,74].

MAPK modulation has also been reported, including extracellular signal-regulated kinase inhibition in lipopolysaccharide-induced hepatic inflammation [73,74]. These non-neural observations provide mechanistic plausibility but require validation in disease-relevant neural models [70].

#### 3.2.2. Modulation of Cytokine Networks and Inflammasome Activation

A hallmark of the anti-inflammatory activity of HT is its ability to modulate cytokine networks and restore immune homeostasis. Excessive production of pro-inflammatory cytokines, including tumor necrosis factor-alpha, interleukin (IL)-1β, IL-6, IL-8, and IL-17, is a characteristic feature of chronic inflammatory disorders and contributes to immune dysregulation, tissue damage, and disease progression. Current evidence indicates that HT significantly attenuates the production and release of these mediators while promoting a more balanced inflammatory profile [6,73,74]. The suppression of cytokine production is largely associated with the inhibition of NF-κB signaling and other redox-sensitive inflammatory pathways, highlighting the close interplay between inflammatory and oxidative mechanisms. Beyond cytokine modulation, HT also regulates inflammasome signaling, a key component of innate immune responses. Among the different inflammasome complexes, the NLRP3 inflammasome has emerged as a major driver of chronic inflammation because of its ability to promote caspase-1 activation, maturation of IL-1β and IL-18, and pyroptotic cell death. Persistent activation of the NLRP3 pathway has been implicated in the pathogenesis of inflammatory bowel disease, neurodegenerative disorders, metabolic dysfunction, and age-related inflammatory conditions. Recent evidence provided by Tang et al. demonstrated that HT suppresses the NLRP3-caspase-1-gasdermin D-IL-1β signaling pathway in DSS-induced colitis, leading to attenuation of mucosal inflammation, preservation of intestinal architecture, and improvement of epithelial barrier function [73]. Because NF-κB provides the priming signal required for inflammasome assembly, the simultaneous inhibition of NF-κB signaling and NLRP3 activation allows HT to target both the initiation and amplification phases of inflammation, disrupting the self-perpetuating cycle that sustains chronic inflammatory responses [70,73].

In a randomized trial, 15 mg/day isolated HT improved selected systemic antioxidant and inflammatory biomarkers in adults with overweight and prediabetes [12]. This is human biological-activity evidence, not confirmation of neural NLRP3 modulation or neuroprotection.

#### 3.2.3. Intestinal Inflammation, Barrier Function, and the Gut Microbiota

The gastrointestinal tract represents one of the most extensively investigated targets of HT. Chronic intestinal inflammation is characterized by disruption of epithelial barrier integrity, excessive cytokine production, oxidative stress, microbial dysbiosis, and increased translocation of microbial-derived inflammatory stimuli. These alterations contribute not only to local tissue injury but also to systemic immune activation through the continuous exposure of the host to pro-inflammatory microbial products. According to Arangia et al., HT exerts protective effects in intestinal and gastrointestinal disorders through the simultaneous modulation of oxidative stress, inflammatory signaling pathways, epithelial barrier function, and gut microbial homeostasis [71]. Recent experimental evidence further demonstrates that HT restores intestinal homeostasis by preserving tight junction proteins, reducing lipopolysaccharide translocation, and limiting the expression of inflammatory genes associated with intestinal injury [73]. In DSS-induced colitis, HT significantly attenuated mucosal damage and improved epithelial barrier integrity, effects that were accompanied by suppression of TLR4/NF-κB signaling and NLRP3 inflammasome activation. Importantly, HT-mediated protection is also associated with significant alterations in gut microbial composition: experimental studies have shown that HT increases the abundance of beneficial bacterial taxa while reducing dysbiosis-associated microorganisms, thereby promoting a microbial environment that favors immune homeostasis and intestinal health [71,73].

Gut–brain relevance remains indirect: microbiota, barrier integrity, and circulating inflammatory signals could influence neuroinflammation, but causal mediation by HT has not been shown in humans [71,72,75].

#### 3.2.4. Hydroxytyrosol, Inflammaging, and Immunometabolic Regulation

Inflammaging links persistent innate-immune activation, oxidative stress, mitochondrial dysfunction, and impaired cellular clearance [6,70]. This framework is relevant to neurodegeneration but is broader than disease-specific evidence.

Reviews propose that HT may couple inflammatory suppression with autophagy-related housekeeping [70]. Because direct neural autophagic-flux data are lacking, this remains a hypothesis rather than an established anti-aging mechanism.

HT may also influence macrophage phenotype through redox and NF-κB signaling, while intestinal-barrier effects could reduce systemic inflammatory stimuli [6,70,71,73]. These links are preclinical and context-dependent.

#### 3.2.5. Crosstalk Between Oxidative Stress and Inflammation

ROS can activate NF-κB and prime NLRP3, while inflammatory signaling increases mitochondrial and immune-cell ROS, creating a feed-forward loop [70,73]. HT can affect both sides through Nrf2/HO-1-related defenses and inflammatory-pathway modulation [6,70].

This crosstalk explains correlated redox and inflammatory changes but does not prove that one pathway mediates the other in vivo. Mechanistic studies should use pathway perturbation and physiologically relevant HT/metabolite concentrations [29,70,73].

#### 3.2.6. Neuroinflammation and Neuroprotective Implications

Chronic glial activation contributes to cytokines, ROS, nitric oxide, synaptic dysfunction, and BBB changes in neurodegenerative disorders [6,70]. HT-specific neural evidence is narrower than the broader inflammation literature.

Reviews synthesize possible effects on microglia, oxidative stress, and BBB integrity [6,8], whereas direct experimental evidence includes reduced lipopolysaccharide- and α-synuclein-induced microglial activation [76]. The latter is more directly relevant to neuroinflammation than colitis or hepatic models.

In aged stressed mice, HT reduced dentate-gyrus neuroinflammation and was associated with neurogenesis and preserved microbial diversity [72]. The design supports an integrated preclinical signal but does not isolate the microbiota as the mediator.

Overall, HT may modulate neuroinflammatory responses, but disease-specific human efficacy and central target engagement remain untested [6,8,72].

The evidence hierarchy is therefore: direct neural support for selected microglial and redox responses; peripheral preclinical support for TLR4/NF-κB, cytokines, and NLRP3; and inferential support for microbiota–gut–brain and inflammaging mechanisms [12,71,73,75,76].

The human trial at 15 mg/day supports systemic biomarker activity only [12]. It should not be used to claim clinical validation of inflammasome inhibition or neuroprotection, for which disease-specific trials are still required [6,8].

The molecular mechanisms underlying the anti-inflammatory effects of HT and their pathophysiological relevance are summarized in Table 2.

### 3.3. Anti-Apoptotic and Mitochondrial Protection

#### 3.3.1. Caspases and Bcl-2/Bax Regulation

In experimental models, HT has been associated with a context-dependent modulation of apoptosis [77,78] and reduced intrinsic apoptotic signaling [25,51]. A non-malignant rat model further showed that HT reversed arsenic-induced changes in Bax, Bcl-2, caspase-3, and cytochrome c [79].

HT also modulated caspase-9/caspase-3 activation and cytochrome c release in a context-dependent manner across preclinical systems [59,80,81,82,83]. These observations do not uniformly indicate cytoprotection but do not establish a disease-specific human mechanism.

#### 3.3.2. Mitochondrial Function

HT preserved mitochondrial membrane potential, respiratory activity, and energy homeostasis in cellular models [84,85].

Effects on fusion/fission and mitochondrial permeability transition have also been reported [16,86,87,88,89], although model and exposure dependence limit direct clinical extrapolation.

#### 3.3.3. Ca^2+^ Homeostasis

HT reduced calcium overload in experimental injury models, thereby limiting calcium-related mitochondrial and oxidative stress [90,91].

Calcium, respiration, and ROS effects are mechanistically connected, but their causal contribution to HT neuroprotection requires pathway-specific validation [91,92,93].

**Figure 2:**
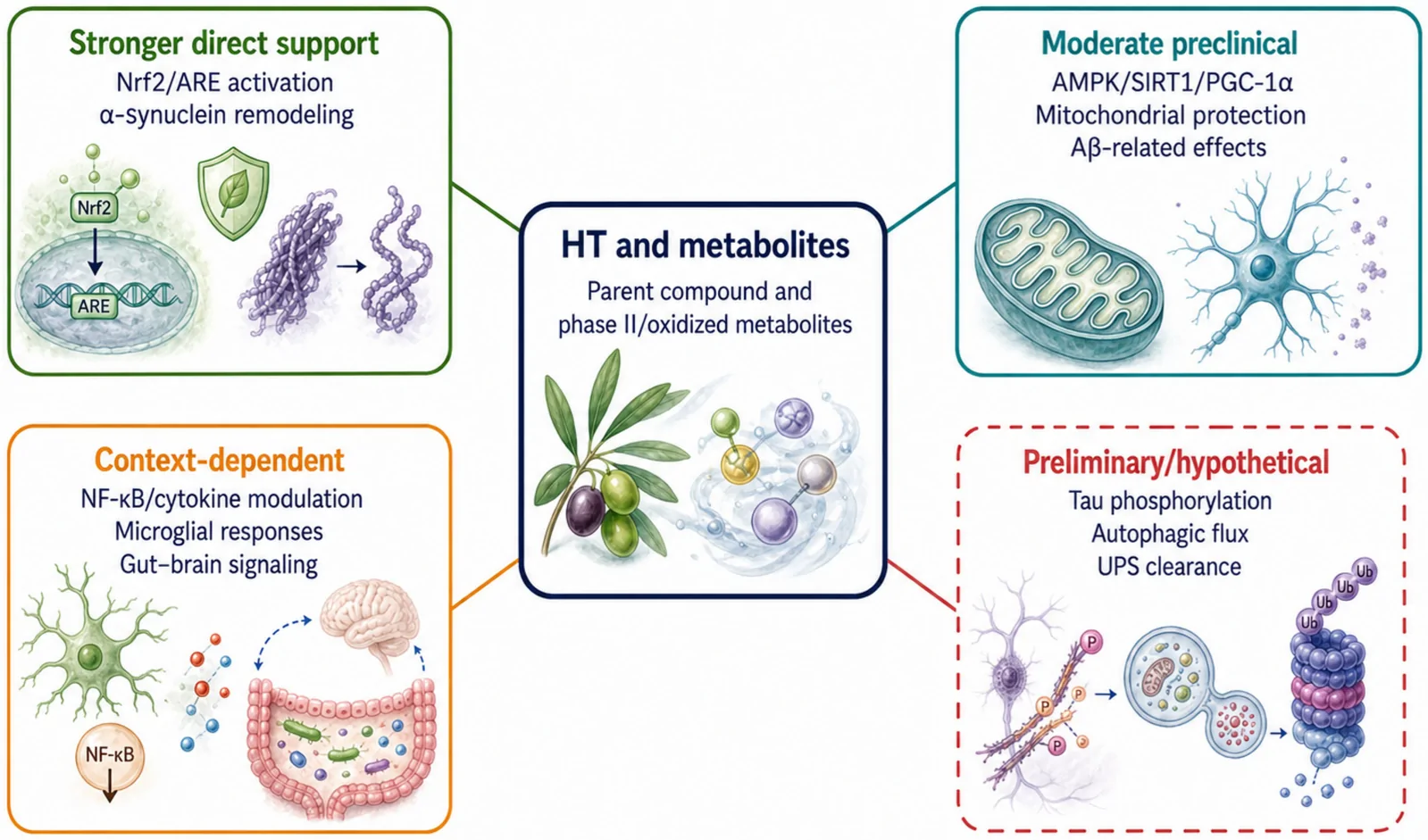
Evidence-weighted integration of neuroprotective mechanisms proposed for HT. Solid categories indicate direct or moderate preclinical support; the dashed category denotes preliminary or hypothetical mechanisms.

The evidence-weighted integration of these neuroprotective mechanisms is shown in Figure 2.

### 3.4. Proteostasis and Protein Aggregation

Proteostasis comprises the interconnected processes that regulate protein folding, trafficking, refolding, and degradation, thereby preserving the integrity of the neuronal proteome. In the brain, disruption of this network favors the accumulation of misfolded and aggregation-prone proteins, particularly Aβ, hyperphosphorylated tau, and alpha-synuclein, which are central to the pathogenesis of AD and PD [8,94,95]. 

An important point in this context is that proteotoxicity is not driven exclusively by mature fibrils or end-stage deposits. Soluble oligomers and intermediate assemblies are often considered the most damaging species, as they can disrupt membranes, alter calcium handling, impair synaptic function, and overload degradative pathways [96]. Accordingly, compounds that reshape aggregation kinetics or alter aggregate conformation may exert meaningful neuroprotection even when they do not completely abolish protein deposition [97,98]. This concept is particularly relevant for HT, whose reported actions often involve remodeling toxic assemblies rather than simply scavenging radicals downstream of protein misfolding [19,99].

#### 3.4.1. β-Amyloid

Aβ aggregation is one of the best-characterized proteotoxic processes in AD. Although insoluble plaques remain the classical histopathological hallmark, soluble Aβ oligomers are now regarded as key mediators of neuronal dysfunction because they impair long-term potentiation, disturb receptor signaling, destabilize membranes, and propagate toxic seeding events [19,94]. Within this framework, HT appears capable of modulating amyloid assembly itself rather than merely attenuating downstream oxidative damage. This distinction is mechanistically important, since compounds that alter aggregate conformation or seeding behavior may reduce toxicity even in the absence of a marked reduction in total amyloid burden. A broader 2025 review on olive polyphenols and amyloid aggregation identifies HT as one of the olive-derived molecules most consistently associated with anti-amyloidogenic activity, especially through interference with early aggregation events and with the structural conversion that stabilizes toxic amyloid species [19,94].

The Aβ evidence therefore combines a direct biophysical study with disease-model outcomes, but no isolated-HT human trial has shown an amyloid biomarker or clinical effect [19,94,100].

#### 3.4.2. α-Synuclein

Among protein targets, α-synuclein has the strongest direct HT-specific support. HT inhibited or remodeled assemblies and reduced associated cellular toxicity [7,76,101]. Nevertheless, much of the in vivo evidence uses *C. elegans* or related tyrosols rather than a mammalian disease model [102,103].

This anti-aggregative effect is now supported by newer structural data. A 2023 computational and biophysical study [101] showed that HT destabilizes α-synuclein oligomers by weakening interchain interactions and reducing β-sheet content within the non-amyloid-β component region and its immediately preceding segment, which together are critical for fibril formation. This finding is important because it provides molecular support for the idea that HT interferes directly with the structural core of α-synuclein self-assembly rather than acting only through secondary cellular effects [101].

Recent work has also expanded the field beyond HT alone to structurally related olive tyrosols. In a 2024 study [102], olive oil tyrosols reduced α-synuclein aggregation in vitro and in a *C. elegans* Parkinson’s model after ingestion, with HT acetate and DOPAC showing particularly strong effects. Even though some derivatives outperformed HT itself in that study, the paper remains highly relevant because it confirms that catecholic olive phenolics as a family can directly suppress α-synuclein aggregation and that this property survives translation into an in vivo model. This also raises an interesting possibility: HT may serve not only as an active molecule itself but also as part of a broader structure-activity landscape in which modifications of the catechol scaffold influence brain permeability and anti-aggregative potency.

The same proteostasis-centered interpretation is reinforced by studies in *C. elegans* showing that HT improves healthspan, reduces α-synuclein accumulation, and protects dopaminergic neurons in Parkinson-like models. Those effects were associated with stress-response factors and with improved proteostatic resilience, suggesting that HT does not simply bind α-synuclein in isolation, but acts within a broader cellular context that determines whether aggregation is tolerated or cleared. Reviews published in 2020–2024 on HT in neurodegeneration cite these data as part of the argument that the compound acts at the intersection of anti-aggregation, anti-inflammatory signaling, and maintenance of protein quality control [65,102,103].

Thus, α-synuclein evidence is mechanistically direct but remains preclinical; its strength should not be confused with readiness for treatment claims [7,76,101,102,103].

#### 3.4.3. Tau Protein Aggregation and Hyperphosphorylation

Tau pathology should be handled more cautiously because the direct evidence for HT is more limited than for α-synuclein and somewhat less developed than for Aβ. Under physiological conditions, tau stabilizes microtubules and supports axonal transport [104]. In disease states, abnormal phosphorylation decreases its affinity for microtubules and increases the tendency of the protein to mislocalize, self-assemble, and form paired helical filaments and neurofibrillary tangles [105]. From the perspective of proteostasis, tau pathology reflects both altered post-translational regulation and failure of degradation pathways to eliminate aberrant tau species efficiently [19,94]. The most direct evidence connecting HT to tau comes from in vitro work showing that olive derivatives, including HT, inhibit tau fibrillization. This remains a cornerstone study because it demonstrated a genuine anti-aggregative effect rather than an indirect reduction in phospho-tau driven by general metabolic improvement. More recent reviews continue to cite this work as the primary basis for considering HT relevant to tau proteopathy [106].

Additional support comes from the 2022 *C. elegans* study using an HT-rich olive extract, which reported reduced tau-associated proteotoxicity alongside effects on Aβ aggregation and oxidative stress. Again, because this was an extract and not pure HT, attribution must remain careful. However, the study still strengthens the argument that HT-containing olive phenolics can attenuate the toxic consequences of tau aggregation in vivo. Importantly, the mechanistic signal pointed toward activation of the skinhead-1 transcription factor (SKN-1)/Nrf2 and heat shock protein 16.2, which links reduced tau proteotoxicity to improved stress adaptation and protein homeostasis [63].

What is less firmly established is whether HT directly reduces tau hyperphosphorylation itself. Some contemporary reviews of olive polyphenols in AD state that HT and related compounds can inhibit tau aggregation, but they usually do not present equally strong direct evidence for selective modulation of tau-directed kinases or phosphatases by HT alone. For that reason, it is more accurate to distinguish between two levels of evidence. There is direct evidence that HT can inhibit tau fibrillization, and there is indirect mechanistic plausibility that it may reduce the intracellular environment favoring pathological tau phosphorylation, because oxidative stress, mitochondrial impairment, and inflammatory signaling all feed into tau dysregulation. The second point is biologically reasonable, but it remains less directly demonstrated than the first [8,94].

HT should therefore be described as a preliminary modulator of tau fibrillization/proteotoxicity, not an established regulator of tau phosphorylation.

#### 3.4.4. Potential but Unconfirmed Modulation of Autophagy and the Ubiquitin-Proteasome System

A discussion of protein aggregation is incomplete without addressing the systems that remove aberrant proteins once they appear. The two major pathways involved are the ubiquitin-proteasome system and the autophagy-lysosomal pathway. In broad terms, the proteasome preferentially degrades short-lived and soluble ubiquitinated proteins, whereas autophagy is more important for large protein aggregates, damaged organelles, and material that exceeds proteasomal capacity. In neurodegenerative disease, dysfunction in either pathway facilitates the persistence of Aβ, α-synuclein, and tau species and increases the probability that transient misfolding will evolve into sustained proteotoxicity. A recent review on the ubiquitin-proteasome system and polyphenol-derived metabolites underlines how closely ubiquitin-proteasome system activity is tied to proteostasis preservation across chronic diseases, including neurodegeneration [107].

For HT, the evidence on these pathways is more indirect than the evidence on direct anti-aggregation, but it is still relevant. In Parkinson-related *C. elegans* models, HT improved health span, reduced α-synuclein burden, and was associated with modulation of heat shock factor 1 and SKN-1 pathways, which are deeply connected to stress adaptation and proteostatic competence. Reviews discussing these data interpret them as supportive of improved protein quality control rather than as a purely anti-inflammatory phenomenon. This matters because the same compound that shifts the folding environment toward lower stress may also reduce the load imposed on degradative systems [65,103]. A more explicit connection between HT and autophagy comes from non-neural models. HT has been shown to activate SIRT1-mediated autophagy in vascular adventitial fibroblasts and, more recently, to enhance SIRT1-dependent autophagy in a 2024 experimental study on skin flap survival. These studies are not neurodegeneration models, so they should not be cited as direct evidence that HT clears Aβ, α-synuclein, or tau via autophagy in the brain. However, they are still valuable because they demonstrate that HT is indeed capable of activating canonical autophagy-related signaling, including SIRT1 and Akt/mechanistic target of rapamycin-associated regulation, in mammalian systems. This substantially strengthens the mechanistic plausibility that similar pathways could contribute to its neuroprotective profile [108,109].

Reviews connect HT with AMPK/SIRT1/PGC-1α and autophagy-related signaling [8,70], providing a testable hypothesis rather than direct disease evidence.

Direct evidence that HT promotes selective ubiquitination or proteasomal disposal of Aβ, tau, or α-synuclein is lacking. Findings involving other proteins or broad polyphenol literature cannot substitute for neural HT-specific experiments [107,110].

**Figure 3:**
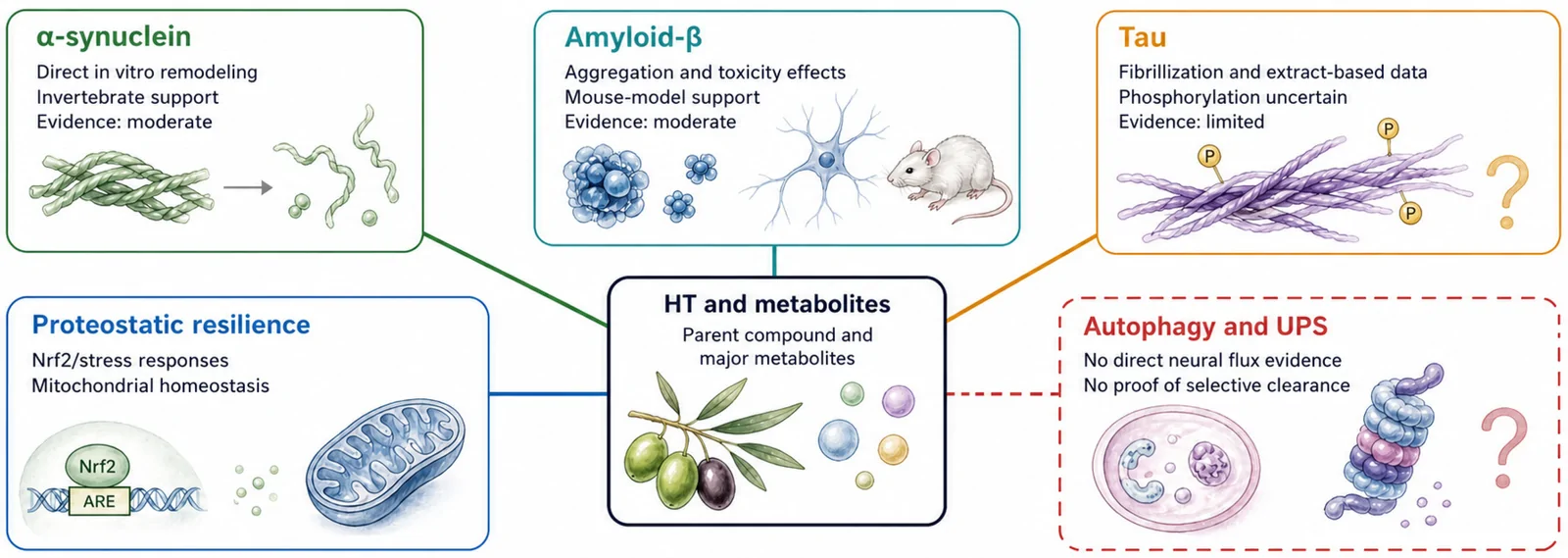
Evidence-weighted relationships between HT, proteostatic resilience, and disease-associated protein aggregation. Dashed pathways indicate plausible but unconfirmed neural clearance mechanisms.

Protein-specific findings and their evidential strength are compared in Table 3 and integrated in Figure 3.

Overall, direct anti-aggregation evidence is strongest for α-synuclein, moderate for Aβ, and limited for tau. Autophagic flux and UPS-mediated aggregate clearance remain unconfirmed in neural disease models [63,76,100,101,102,103,104,105,106,107,108,109,110].

## 4. Evidence in Specific Neurodegenerative Disorders

### 4.1. Alzheimer’s Disease

AD is the most extensively studied disorder for HT, but evidence remains predominantly preclinical [13,65,94,115]. Relevant processes include oxidative/mitochondrial stress, neuroinflammation, Aβ aggregation, tau dysregulation, and synaptic failure [19,94,115].

HT-related redox effects are most plausibly mediated through Nrf2/ARE and mitochondrial adaptation rather than stoichiometric radical scavenging [8,16,94,116,117,118,119].

In vitro studies report reduced Aβ-related toxicity and improved mitochondrial or redox outcomes [16,19,63,87,100]. These models do not establish achievable human brain concentrations.

Animal studies provide additional support, although their interpretation requires caution [65,111]. In a TgCRND8 mouse model of Aβ deposition, dietary supplementation with HT improved cognitive performance and reduced both Aβ42 and pyroglutamate-modified Aβ plaque burden [111]. This study is particularly relevant because it links HT exposure not only to molecular or histological changes, but also to functional cognitive improvement [111]. The reduction in plaque area and number suggests that HT may interfere with amyloid pathology in vivo, although the precise mechanisms remain difficult to separate from its broader antioxidant and anti-inflammatory actions [19,65]. In this sense, the study supports the view that HT may act upstream and downstream of amyloid accumulation, by modulating both the formation of toxic assemblies and the cellular environment that determines their toxicity [19,94,111].

A complementary perspective comes from amyloid precursor protein/presenilin 1 mouse models, where HT has been reported to mildly improve cognitive function without clearly modifying amyloid precursor protein processing [112]. This distinction is important because it suggests that cognitive benefits may occur even in the absence of a major effect on amyloid production itself. In other words, HT may improve neuronal resilience, synaptic function, mitochondrial activity, or redox balance without necessarily acting as a classical amyloid-lowering agent [13,16]. This interpretation is consistent with the broader concept that AD progression depends not only on amyloid burden, but also on the capacity of neural systems to tolerate proteotoxic stress, maintain energy metabolism, and limit neuroinflammatory amplification [16,19,94].

Tau evidence is weaker: olive phenols or HT-rich extracts may reduce aggregation/proteotoxicity, but isolated HT has not been shown to selectively regulate tau phosphorylation in AD models [19,63,65,94,114].

As described in Section 3.4.3, the study using an olive-derived extract 20% rich in HT in *Caenorhabditis elegans* is especially useful in this context because it connects several AD-related mechanisms in the same experimental framework. This extract reduced Aβ aggregation and oxidative stress and showed mechanistic involvement of SKN-1/Nrf2 and heat shock protein 16.2 pathways [63]. Although this was not a pure HT intervention, it supports the idea that HT-containing olive phenolics may improve stress-response capacity and protein homeostasis in vivo [63,114]. At the same time, the use of a mixed extract means that the effects cannot be attributed exclusively to HT. This limitation should be explicitly acknowledged because it is a recurring issue in the olive polyphenol literature [63,94,114].

Mitochondrial protection is relevant because bioenergetic failure can precede or amplify amyloid/tau pathology; nevertheless, most HT evidence comes from metabolic or oxidative-stress models rather than AD-specific experiments [16,59,94,120,121].

The human evidence is more indirect and should be clearly separated from the preclinical findings [65,94,115,122]. Trials with high-phenolic extra-virgin olive oil in individuals with mild cognitive impairment suggest potential cognitive benefits and improvements in AD-related biological markers, including oxidative and nitrative stress parameters, blood–brain barrier integrity, and functional connectivity [115,123,124,125]. However, these studies do not isolate HT as the active compound. Extra-virgin olive oil contains multiple phenolic and lipid components, including oleuropein derivatives, oleocanthal, tyrosol derivatives, and monounsaturated fatty acids, which may act synergistically [122,124]. Therefore, these studies are highly relevant for the translational context of olive-derived phenolics, but they cannot be interpreted as direct clinical evidence that HT alone modifies AD progression [94,115].

Consequently, AD support is moderate and preclinical, strongest for redox/mitochondrial and Aβ-related outcomes, weaker for tau, and indirect in humans. Trials should use standardized HT, exposure biomarkers, plasma/CSF Aβ42/40 and phosphorylated tau, neurofilament light chain, imaging, and cognitive outcomes [40,55,111].

### 4.2. Parkinson’s Disease

PD combines dopaminergic neuronal loss, α-synuclein pathology, oxidative/mitochondrial stress, and microglial activation [126,127]. Standard symptomatic therapies include dopamine replacement and monoamine oxidase-B (MAO-B) inhibitors; HT is an experimental candidate, not an alternative treatment.

Several in vitro studies have examined the direct protective effects of HT on dopaminergic and glial cell models relevant to PD. Gallardo-Fernández et al. showed that HT decreases both lipopolysaccharide- and α-synuclein-induced microglial activation, attenuating the release of pro-inflammatory mediators implicated in the propagation of dopaminergic cell loss [76]. In dopaminergic-lineage cell lines, Yu et al. demonstrated that HT activates the Nrf2 pathway to induce phase II detoxifying enzymes, including heme oxygenase-1, thereby protecting against dopamine- and 6-OHDA-induced cytotoxicity [128], while Manzoor et al. reported a synergistic neuroprotective effect between endogenously produced HT and synaptic vesicle proteins against salsolinol, a dopamine-derived neurotoxin, in PC12 cells [129]. Using an in vitro BBB co-culture model comprising human cerebral microvascular endothelial D3 and human neuroblastoma cells, Mursaleen et al. showed that micellar nanocarriers of HT are protective against Parkinson’s-related oxidative stress, improving delivery relative to free HT [56]. Notably, in vitro aggregation assays by Hernández-García et al. showed that hydroxytyrosol acetate (HTA), a naturally occurring HT ester, completely blocked α-synuclein fibril formation, outperforming both HT and the dopamine metabolite DOPAC under identical experimental conditions [102].

In vivo evidence spans both rodent and invertebrate models of PD, covering the principal chemically induced paradigms used in the field. In mice, Pathania et al. combined in silico target prediction with a 1-methyl-4-phenyl-1,2,3,6-tetrahydropyridine-induced model to show that HT restores striatal dopamine levels and improves motor performance, implicating a multitarget mechanism rather than a single molecular interaction [130]. Siracusa et al. reported that Hidrox^®^, a standardized HT-rich olive-derived extract, exerts anti-inflammatory and antioxidant effects in a rotenone-induced mouse model [131]. In rats, Pérez-Barrón et al. compared HT with its acetate and nitro-derivatives in a 1-methyl-4-phenylpyridinium (MPP+) model and found that all three attenuated oxidative damage, though with differing potency depending on the derivative [132]; in a related study, the same group showed that HT inhibits monoamine oxidase (MAO) isoforms and thereby prevents MPP+-induced neurotoxicity in vivo, directly linking its antioxidant and enzyme-inhibitory actions [133]. In the invertebrate *Caenorhabditis elegans*, Hernández-García et al. showed that dietary intake of HT and, more markedly, HTA reduced α-synuclein aggregation in vivo by up to 76.2%, with HTA also showing the highest permeability through brain-lipid membranes among the compounds tested [102]. Brunetti et al. similarly reported that HT and oleuropein aglycone maintain healthspan and prevent Parkinson’s-like phenotypes, including dopaminergic neurodegeneration and impaired motility, in *C. elegans* [103].

BBB transport percentages and nanocarrier findings are evaluated centrally in Section 2.2.4. They show model-specific transport capacity, not pharmacologically relevant human brain exposure [10,54,55,56].

#### 4.2.1. Molecular Mechanisms

Direct interference with α-synuclein aggregation is the most disease-specific mechanism [7,76,101,102]. HT acetate showed greater activity than parent HT in vitro and in *C. elegans*, but requires confirmation in mammalian pharmacokinetic and PD models [102].

Nrf2-related cytoprotection and antioxidant effects are supported in dopaminergic cells and toxin models [128,130,131,132]. Findings with Hidrox^®^ or derivatives cannot be assigned exclusively to HT [131,132].

HT also reduced microglial activation in vitro [76]. Broader anti-inflammatory effects are plausible, but their relative contribution to dopaminergic protection in vivo is unresolved [70,131,134].

A rat MPP+ study using 1.5 mg/kg intravenous HT reported altered MAO-A/MAO-B activity and neuroprotection [133]. It did not establish MAO-B selectivity, IC50, reversibility, oral exposure, or human central target engagement. Unlike approved selegiline, rasagiline, or safinamide, HT therefore cannot be considered a validated MAO-B inhibitor or substituted for PD medication [127,133].

Direct evidence that HT restores autophagic flux or mitophagy in PD models is absent; this remains a testable hypothesis [70].

The PD evidence hierarchy is direct preclinical support for α-synuclein remodeling and selected Nrf2/microglial effects; toxin-model support for functional outcomes; preliminary MAO evidence; and unconfirmed autophagy. Table 4 summarizes the models.

#### 4.2.2. Clinical Evidence

No completed disease-specific trial has tested isolated HT in PD. A ClinicalTrials.gov search updated on 24 August 2026 identified no PD-specific isolated-HT trial; the recruiting MCI study NCT07672938 tests olive-juice polyphenols plus Mediterranean-diet advice rather than isolated HT (https://clinicaltrials.gov/study/NCT07672938, accessed on 10 July 2026). Current human studies therefore support safety, pharmacokinetics, or systemic biomarkers, not PD efficacy [8,12,55,102].

### 4.3. Other Disorders: Preliminary Evidence

Evidence beyond AD and PD is sparse, usually indirect, and should not be presented at equivalent depth or certainty.

For amyotrophic lateral sclerosis, proposed benefits are extrapolated from general redox, mitochondrial, and inflammatory mechanisms rather than direct isolated-HT efficacy [135,136,137,138].

No disease-specific clinical evidence currently supports HT for amyotrophic lateral sclerosis.

For Huntington’s disease, the cited 3-nitropropionic-acid rat study tested olive oil, not purified HT; it supports olive-matrix plausibility rather than HT-specific efficacy [20,139].

No isolated-HT Huntington trial or disease-modifying evidence was identified.

For mild cognitive impairment (MCI), cognitive, biomarker, and BBB signals derive mainly from phenolic-rich olive-oil interventions [123,124,125], while experimental HT findings remain preclinical [112,140].

A recruiting registered MCI trial (NCT07672938) evaluates olive-juice polyphenols at two doses with Mediterranean-diet advice, not isolated HT (https://clinicaltrials.gov/study/NCT07672938, accessed on 10 July 2026).

Accordingly, these disorders provide hypothesis-generating evidence and translational context, but no basis for HT-specific treatment claims.

## 5. Clinical Evidence and Human Studies

Human evidence remains early and heterogeneous. Most studies test pharmacokinetics, safety, vascular/metabolic outcomes, or systemic redox markers rather than neurodegenerative disease [8,12,141]. Intervention classes are separated below to prevent causal over-attribution.

Isolated HT, HT-enriched olive oil, phenolic-rich foods, and mixed supplements answer different questions. Positive results from a matrix cannot be assigned to HT alone.

### 5.1. Isolated Hydroxytyrosol Interventions

Human intervention studies with isolated or near-isolated HT are still relatively scarce, and only a minority address outcomes directly relevant to brain health. Much of the earlier clinical work focused on bioavailability, safety, and oxidized low-density lipoprotein (LDL)-related endpoints rather than cognition or neurological function. Recent reviews therefore emphasize that the translational clinical literature for HT remains broader in the cardiometabolic field than in neurology, despite the strong mechanistic rationale for neuroprotection [8,10]. Among the available human studies, the most informative data for isolated HT come from randomized controlled trials evaluating cardiometabolic and oxidative outcomes rather than direct neurological endpoints. One of the most relevant recent placebo-controlled studies is the 2025 randomized, double-blind, parallel trial by Moratilla-Rivera and colleagues [12] in adults with overweight and prediabetes. Participants received 15 mg/day of HT for 16 weeks, and the primary outcome was oxidized LDL. Compared with placebo, HT significantly reduced oxidized LDL, protein carbonyls, and 8-hydroxy-2′-deoxyguanosine (8-OHdG), while also preventing declines in total antioxidant status and glutathione peroxidase activity. A modest anti-inflammatory signal was also observed through lower IL-6 concentrations. Importantly, however, no significant effects were found on lipid profile, anthropometry, sleep, mental well-being, or physical capacity. This trial is highly relevant because it provides well-controlled evidence that isolated HT can improve systemic oxidative and inflammatory status in an at-risk population, yet it simultaneously illustrates the current translational gap: the measured benefits are largely biochemical and preventive rather than overtly neurological. 

An even more disease-oriented, though methodologically weaker, example is the 2025 open-label pilot study in pediatric mitochondrial disease [142]. In that trial, HT was administered daily for 12 months, after which participants were randomized either to continue supplementation or to discontinue it. Outcome measures included the International Paediatric Mitochondrial Disease Scores, biochemical variables, magnetic resonance imaging (MRI)-based assessments, and quality-of-life measures. The clearest reported effect was improvement in health-related quality of life, with a possible signal in a subgroup of patients with mitochondrial encephalomyopathy, lactic acidosis, and stroke-like episodes. Tolerability was good, and no adverse effects were reported. Nevertheless, the study enrolled only nine participants, lacked placebo blinding, and used a withdrawal-type design because a matching placebo was unavailable. For those reasons, it is best interpreted as proof of feasibility and tolerability, with preliminary clinical signal, rather than as confirmatory evidence of efficacy. Still, it is one of the few recent studies in which HT itself has been tested in a human condition with neurological and mitochondrial relevance. 

Thus, isolated HT is biologically active in humans, but no adequately powered AD, PD, or other proteinopathy trial has established cognitive, motor, imaging, fluid-biomarker, or disease-progression efficacy.

### 5.2. Hydroxytyrosol-Enriched Olive Oil

HT-enriched and high-phenolic oils offer realistic delivery but contain coactive lipids and phenols, limiting compound-specific attribution.

In patients with chronic coronary disease, Ikonomidis and colleagues reported in 2023 that HT-enriched olive oil improved endothelial and arterial function and was accompanied by reductions in malondialdehyde, oxidized LDL, triglycerides, proprotein convertase subtilisin/kexin type 9, and C-reactive protein [143]. The authors concluded that the vascular benefits were likely mediated, at least in part, by lower oxidative and inflammatory burden. Although these were cardiovascular rather than neurological endpoints, the study is clinically relevant to neuroprotection because endothelial function, arterial stiffness, oxidative stress, and inflammation are tightly linked to cerebrovascular aging and cognitive decline. It therefore supports the idea that HT-containing olive interventions may act on systemic pathways that are highly relevant to brain resilience. 

A shorter-term crossover study published the same year examined an olive oil extract enriched with HT in patients with chronic coronary artery disease over 30 days [144]. The intervention was associated with only a trend toward improvement in left ventricular diastolic function and pulse wave velocity, and the authors explicitly noted that longer studies would be needed to clarify the clinical impact. This study is useful precisely because it tempers the optimism generated by more positive reports: even when the biological rationale is plausible, short interventions may be underpowered to produce clear functional gains. 

Further evidence comes from the 2025 pilot randomized clinical trial in healthy and post-myocardial infarction elderly adults consuming different olive oils for 26 weeks [145]. Participants receiving high-tyrosol/HT extra-virgin olive oil showed enhancement of antioxidant-related measures, including changes in malondialdehyde, ferric reducing antioxidant power, and high-density lipoprotein-associated parameters such as paraoxonase-1 and lecithin-cholesterol acyltransferase activity. Again, this does not directly establish neuroprotection, but it supports a clinically meaningful redox-modulating effect of olive matrices rich in tyrosol/HT in an elderly population, which is highly relevant to the aging brain. At the same time, the small pilot nature of the study and the combined phenolic exposure make causal attribution to HT alone impossible. 

### 5.3. Mixed Formulations and Foods

HT-enriched bread improved metabolic and inflammatory outcomes in adults with type 2 diabetes [146], while an HT-plus-punicalagin supplement improved dyslipidaemia [147]. These findings support formulation activity but not isolated HT or neurological efficacy.

Overall, trials using enriched oils and HT-containing foods indicate that HT can be clinically active in realistic dietary matrices and may improve vascular, oxidative, inflammatory, and metabolic parameters that are relevant to long-term brain health [143]. Nevertheless, these studies also illustrate a persistent attribution problem: once HT is embedded in a phenolic-rich olive matrix or a multi-component nutraceutical, it becomes difficult to separate its contribution from that of coexisting olive phenols, oleic acid, or other added compounds. 

### 5.4. Cognitive and Oxidative Biomarkers

The most neurorelevant human signals come from extra-virgin olive oil in MCI, not isolated HT.

In the pilot randomized clinical trial by Tsolaki et al. [125], individuals with mild cognitive impairment consumed high-phenolic early-harvest extra-virgin olive oil, moderate-phenolic extra-virgin olive oil, or received Mediterranean diet advice. The study reported improvements in several cognitive domains, including global cognition and other neuropsychological measures, in the high-phenolic olive oil group. This trial is often cited as one of the strongest human demonstrations that phenolic-rich olive oil may support cognition in prodromal neurodegeneration. However, the intervention was not an HT-only exposure, and the phenolic profile also included other potentially active compounds such as oleocanthal and oleuropein derivatives. For that reason, the study is highly relevant to the translational argument but does not isolate the role of HT. 

A 2022 randomized controlled trial by Kaddoumi and colleagues [124] extended this line of evidence by showing that extra-virgin olive oil, compared with refined olive oil, reduced BBB permeability in people with mild cognitive impairment, as assessed by contrast-enhanced MRI, and improved functional connectivity. Both olive oils improved some cognitive and blood biomarker measures, but extra-virgin olive oil uniquely improved BBB integrity. From the standpoint of translational neuroprotection, this is a very important result because BBB dysfunction is increasingly recognized as an early contributor to AD and cognitive decline. Yet once again, the study tested extra-virgin olive oil as a complex matrix rather than HT in isolation, so the findings are best interpreted as evidence for phenolic-rich olive interventions, not for single-compound HT efficacy. 

Oxidative and nitrative biomarker studies reinforce the same pattern. In a 2021 biomarker analysis derived from the same trial [123], extra-virgin olive oil consumption in mild cognitive impairment was associated with reduced DNA oxidative damage, lower poly(ADP-ribose) polymerase 1 levels, attenuation of nitrative stress, and changes in circulating Alzheimer-related markers such as Aβ1-42. These findings are particularly relevant because they connect human olive-oil intervention with molecular readouts that are plausibly linked to neurodegenerative pathophysiology. However, they still reflect the action of a phenolic-rich oil rather than HT alone and therefore should be cited as supportive translational evidence rather than definitive HT-specific proof. 

As described in Section 5.1 and Section 5.2, outside the cognitive impairment field, oxidative biomarker modulation is one of the most reproducible signals in human HT research. The 2025 trial in overweight adults with prediabetes [12] demonstrated significant reductions in oxidized LDL, protein carbonyls, and 8-OHdG with isolated HT, while the enriched-oil trials in chronic coronary disease and elderly post-myocardial infarction participants also reported decreases in lipid peroxidation markers and improvements in antioxidant-related parameters. This repeated convergence on redox-related biomarkers is important because oxidative damage is one of the mechanistic bridges between systemic vascular dysfunction and neurodegeneration [143,145]. Nevertheless, biomarker responsiveness is not equivalent to clinical neuroprotection, and the field still lacks studies demonstrating that these biochemical improvements translate into slower cognitive decline or reduced incidence of neurodegenerative disease. 

Current human evidence therefore supports systemic biological activity and a matrix-level cognitive signal, but not disease-modifying efficacy of isolated HT [8,115]. The recruiting MCI trial NCT07672938 should clarify olive-polyphenol effects, not HT-specific causality (https://clinicaltrials.gov/study/NCT07672938, accessed on 10 July 2026).

### 5.5. Limitations of the Current Clinical Evidence

The first major limitation is sample size. Many of the most interesting studies are pilots or exploratory trials. The mitochondrial disease study enrolled only nine participants, the mild cognitive impairment trial with MRI outcomes included only around two dozen participants completing the protocol, and even some of the enriched oil cardiometabolic studies remained relatively small. Small samples reduce statistical power, increase the risk of unstable effect estimates, and make subgroup findings difficult to interpret confidently [124,142]. 

The second limitation is intervention heterogeneity. “HT exposure” in the human literature can mean purified HT capsules, synthetic HT, HT-enriched olive oil, high-phenolic extra-virgin olive oil, HT-enriched bread, or mixed polyphenol supplements. These interventions differ not only in the amount of HT delivered, but also in matrix composition, coexisting phenolics, lipid context, absorption kinetics, and metabolic fate. This heterogeneity makes it difficult to compare studies directly and to determine whether positive outcomes are attributable to HT itself, to the matrix, or to a synergy among several constituents [10,146,147]. 

The third limitation is endpoint heterogeneity. Some trials focus on oxidized LDL, 8-OHdG, protein carbonyls, or inflammatory cytokines; others emphasize endothelial function, pulse wave velocity, glucose control, MRI BBB permeability, functional connectivity, or neuropsychological scales. While this breadth shows that HT may act on multiple biological domains, it also fragments the evidence base and hinders quantitative synthesis. More importantly, very few studies assess hard neurological endpoints such as conversion from mild cognitive impairment to dementia, progression rates in neurodegenerative disease, or validated disease-specific fluid biomarkers over sufficiently long follow-up [12,124]. 

A fourth limitation concerns trial design. Some studies are open-label, some use crossover designs with relatively short washout periods, and some rely on short intervention periods that may be too brief to capture meaningful changes in cognition or disease trajectory. The pediatric mitochondrial disease study explicitly adopted an open-label withdrawal design because a matching placebo was unavailable, and the short-term enriched-olive-oil cardiovascular study itself concluded that longer trials were needed to delineate the true effect. These are not fatal flaws, but they do reduce inferential strength [142,144]. 

A fifth limitation is population mismatch. Many studies have been conducted in overweight individuals, patients with cardiometabolic risk, people with chronic coronary disease, or adults with type 2 diabetes. These populations are highly relevant to aging and vascular contributions to cognitive decline, but they are not equivalent to patients with AD, PD, dementia with Lewy bodies, or other target neurodegenerative conditions. Therefore, a favorable effect on systemic oxidative stress or vascular biomarkers should not be interpreted as direct evidence of efficacy against neurodegenerative proteinopathies without further bridging studies [12,143]. 

Finally, compliance and exposure verification are not always equally rigorous. One strength of the 2025 isolated-HT trial was the use of urinary HT-3′-sulfate to confirm compliance. Similar biomarker-based confirmation is not consistently available across the wider literature, and this becomes especially important for compounds such as HT whose metabolism is rapid and matrix-dependent. In future trials, systematic quantification of urinary or plasma metabolites would strengthen causal interpretation considerably [10,12]. 

### 5.6. Quantitative Dose Comparison and Preclinical Extrapolation

In vitro concentrations, animal mg/kg doses, and human mg/day intakes are different dimensions and cannot be equated without pharmacokinetic assumptions. Table 5 compares representative exposures.

As described in Section 5.1, in the randomized, double-blind, placebo-controlled parallel trial by Moratilla-Rivera et al., adults with overweight and prediabetes received 15 mg/day of isolated HT for 16 weeks, resulting in significant reductions in oxidized LDL, protein carbonyls, 8-hydroxy-2′-deoxyguanosine, and IL-6, with compliance confirmed by urinary HT-3′-sulfate [12]. This study is particularly informative because it combines a clearly defined daily dose, a relatively long intervention period, placebo control, and exposure verification. However, its endpoints were mainly oxidative and inflammatory rather than neurological, so it supports biological activity and dose feasibility more than direct neuroprotective efficacy.

A somewhat different perspective emerges from the longer-term trial by Fytili et al., in which women with overweight or obesity received 5 mg/day or 15 mg/day of HT for 6 months [148]. Unlike Moratilla-Rivera et al. [12], this study focused primarily on body weight, fat mass, and urinary metabolomics rather than oxidative LDL-related endpoints. Taken together, these two trials illustrate an important point: even when similar nominal doses are used, the interpretation of clinical relevance depends heavily on the population studied, the intervention length, and the primary outcomes selected.

An additional layer of complexity appears in acute exposure studies. In the intervention by Bender et al., healthy volunteers received olive-derived liquid food supplements providing 30.6 mg or 61.5 mg of HT in a single 25 mL dose, which led to clear absorption, detectable metabolite formation, and acute reductions in lipid oxidation biomarkers [40]. These findings are highly valuable from a pharmacokinetic perspective, since they show that moderate single oral doses can produce measurable short-term biological effects. At the same time, acute postprandial exposure cannot be directly equated with chronic supplementation, and therefore such studies cannot by themselves define the dose range required to reproduce the sustained mechanisms described in preclinical neuroprotection models.

Food interventions cannot be compared by nominal HT amount alone because matrix and intake pattern alter exposure [146].

Likewise, HT-enriched oil outcomes are product-specific and not equivalent to isolated-HT dosing [143].

For an HT molecular mass of 154.16 g/mol, 10–50 μM corresponds to 1.54–7.71 mg/L. It does not correspond to 1.5–7.5 mg/kg; conversion to a body-mass dose requires route, volume of distribution, absorption, and bioavailability.

Across studies, chronic isolated doses are commonly 5–15 mg/day, acute oral exposures can be higher, and preclinical studies use concentration- or body-mass-based regimens. These should remain separate in evidence synthesis [12,40,49,133,148].

Human studies report generally good tolerability, including no adverse events at 15 mg/day for 16 weeks [12,141], but safety and biomarker activity do not establish neurodegenerative efficacy.

## 6. Translational Gaps and Future Directions

The principal translational gap is whether preclinical mechanisms occur in humans at achievable exposure and improve validated neurological outcomes [8,12,13,55,94,115,141].

Dose comparisons should use pharmacokinetic exposure rather than equating μM, mg/kg, and mg/day. Matrix, route, timing, and metabolism must be reported, as summarized in Table 5 [10,12,40,49,133,141].

A second unresolved issue is whether the biologically active forms of HT reach the central nervous system at concentrations sufficient to reproduce the effects observed in vitro. HT is rapidly absorbed but extensively metabolized, and systemic circulation is dominated by glucuronide, sulfate, methylated, and oxidized derivatives rather than by the free parent compound [10,40]. This means that many in vitro studies using unconjugated HT may not fully represent the molecular species to which human tissues are exposed. Future studies should therefore move beyond the parent compound and evaluate the neurobiological activity of the main circulating metabolites, including their ability to cross the BBB, accumulate in relevant brain regions, undergo local deconjugation, or act through peripheral mechanisms that indirectly influence brain health [10,40,53,55].

No study has quantified HT or its major metabolites in human brain or cerebrospinal fluid after oral supplementation. Paired plasma/CSF pharmacokinetics, validated deconjugation methods, and pharmacodynamic readouts are priorities [10,52,55].

Formulation development represents another important future direction. Conventional oral HT may be limited by rapid metabolism, short persistence of the free molecule, and variable matrix-dependent absorption [10,40]. Nanocarriers, lipid-based systems, encapsulated formulations, prodrugs, and chemically modified derivatives could theoretically improve stability, intestinal absorption, BBB transport, and tissue retention [149,150]. However, these strategies should not be presented as clinically validated solutions yet. At present, they are best interpreted as promising technological approaches that require careful evaluation of safety, pharmacokinetics, brain delivery, and comparative efficacy against standard HT formulations [141,149,150]. Importantly, improved delivery should only be considered meaningful if it translates into stronger modulation of validated neurodegenerative biomarkers or better clinical outcomes [8,141].

Another major limitation is the absence of adequately powered RCTs specifically designed for neurodegenerative disease. Most available human studies have focused on cardiometabolic risk, vascular function, oxidative stress, or mild cognitive impairment, and many have used complex olive-derived matrices rather than isolated HT [8,124,125,141]. Future RCTs should be designed according to the biological question being tested. If the aim is to determine the specific effect of HT, trials should use standardized isolated HT or well-characterized HT-enriched formulations with precise dose reporting and metabolite-based compliance assessment [10,40,141]. If the aim is to evaluate the broader effect of olive phenolic matrices, this should be stated clearly, avoiding attribution of the results to HT alone [8,124,125].

The choice of target population is also critical. Preventive trials in healthy adults may require very long follow-up periods and large sample sizes to detect meaningful neurological effects. In contrast, individuals with mild cognitive impairment, early AD, prodromal PD, vascular cognitive impairment, or high cardiometabolic risk may represent more suitable populations for detecting short- to medium-term biological signals [8,13,141]. Nevertheless, these groups should not be treated as interchangeable. The mechanisms driving AD, PD, amyotrophic lateral sclerosis, Huntington’s disease, and vascular cognitive decline differ substantially, and the expected contribution of oxidative stress, protein aggregation, mitochondrial dysfunction, or BBB impairment may vary across disorders [141]. Future studies should therefore match the target population with the most plausible mechanism of action of HT [8,13,151].

A further priority is the use of validated and disease-relevant biomarkers. General oxidative stress markers, such as oxidized low-density lipoprotein, malondialdehyde, protein carbonyls, or 8-hydroxy-2′-deoxyguanosine, are useful to demonstrate systemic biological activity, but they are not sufficient to establish neuroprotection [151,152]. Future trials should combine systemic redox and inflammatory markers with neurological biomarkers such as Aβ, phosphorylated tau, total tau, neurofilament light chain, α-synuclein-related markers, neuroimaging outcomes, BBB permeability measures, and standardized cognitive or motor assessments [124]. This multimodal approach would help determine whether HT acts only on peripheral oxidative burden or whether it can also influence central pathways directly involved in neurodegeneration [8,152,153].

Personalized approaches may also be relevant. Interindividual variability in HT metabolism, gut microbiota composition, baseline oxidative status, BBB integrity, cardiometabolic health, age, sex, medication use, and genetic background may influence both exposure and response [10,40,154]. In particular, redox-related genetic variability, apolipoprotein E genotype, mitochondrial vulnerability, and differences in phase II metabolism could contribute to heterogeneous treatment effects [10,153]. Future studies should consider stratified analyses or biomarker-guided designs to identify responder profiles rather than assuming a uniform effect across all participants [40,153,154].

Finally, future research should clearly distinguish between three related but different concepts: HT as an isolated bioactive compound, HT as part of an olive-derived phenolic mixture, and HT as one contributor within a broader Mediterranean dietary pattern [8,115,141]. This distinction is essential for mechanistic interpretation, regulatory positioning, and clinical translation. Positive findings from extra-virgin olive oil or Mediterranean diet interventions are highly relevant to public health, but they cannot be used as direct evidence of isolated HT efficacy [8,94,141]. Conversely, trials with purified HT may clarify compound-specific effects but may not reproduce the synergistic matrix effects that occur with olive oil consumption. Both approaches are valuable, but they answer different scientific questions [10,94,115,141].

Until standardized exposure and disease-specific efficacy data are available, HT should be considered an experimental neuroprotective candidate with human systemic biological activity, not an established preventive or disease-modifying intervention [8,10,40,141,154].

## 7. Safety and Regulatory Aspects

As noted in Section 4.2.2, the expanding use of HT in functional foods, nutraceuticals, cosmetics, and emerging pharmaceutical formulations has increased interest in its long-term safety and regulatory status. Current toxicological evidence consistently indicates that HT is neither genotoxic nor mutagenic under standard experimental conditions, with negative findings obtained in Ames tests, in vitro micronucleus assays, and comet assays across different experimental models [155,156]. Likewise, acute, subchronic, reproductive, and developmental toxicity studies have not identified relevant adverse effects, and systematic reviews consistently report a favorable metabolic and toxicological profile in both experimental animals and humans [155,157]. On this basis, EFSA concluded that synthetic HT was safe under the proposed food uses and use levels, using a no-observed-adverse-effect level (NOAEL) of 50 mg/kg body weight/day to calculate margins of exposure [50]. Separately, an in vitro genotoxicity assessment and literature review considered an olive juice dry extract standardized to 20% HT (OE20HT) safe at doses up to 2.5 mg/kg body weight/day, while regulatory conditions and permitted uses differ across the EU, US, and China [155,157]. Available human trials likewise report no clinically relevant adverse effects after oral HT supplementation, although most are of short duration and modest sample size [155].

Although these findings support the overall safety of HT, some experimental studies indicate that its biological effects may vary according to dose, formulation, and tissue distribution. For example, polyphenol-rich olive oil administered within the range covered by the EFSA olive-oil polyphenol health claim reinforced antioxidant status in blood, brain, muscle, and intestine of rats, but induced signs of oxidative stress in spleen, pancreas, liver, and heart, with no discernible effect in lung, colon, or kidney [158]. Rather than questioning the safety of HT, these findings underscore the importance of tissue-resolved toxicological evaluation, particularly when extrapolating nutraceutical safety margins to chronic, long-term human exposure.

Beyond its own favorable safety profile, HT and its derivatives have also shown protective activity against toxicity from unrelated agents, reversing zearalenone-induced senescence markers in zebrafish [159], protecting microglia from bisphenol S toxicity via cytochrome P450 1A1 modulation [9] and, in ester form, acting as anti-amyloid neuroprotective candidates for AD without evidence of relevant in vivo toxicity [160]. Newer delivery formats are also being safety-tested directly: nanoencapsulation of an HT-rich extract in proniosomes raised, rather than lowered, the safety margin in a zebrafish embryo assay [161], and olive pomace-derived HT/oleuropein extracts intended for ophthalmic use passed genotoxicity and six-month stability testing under International Council for Harmonisation-aligned conditions [156].

Taken together, the absence of genotoxicity, mutagenicity, and clinically relevant adverse effects, together with EFSA’s conclusion of safety under the proposed uses and use levels, supports the current safe use of HT in food supplements and functional foods. Nevertheless, the tissue-specific variability documented under real-life exposure conditions, the comparative scarcity of long-term human data, and the emergence of novel esters and nanoencapsulated formulations indicate that continuous toxicological evaluation and appropriate regulatory oversight remain warranted as HT’s therapeutic and nutraceutical applications continue to expand [155,157,158].

## 8. Conclusions

HT has a coherent multitarget preclinical profile. Support is strongest for Nrf2/ARE activation and direct α-synuclein remodeling, moderate for AMPK/SIRT1/PGC-1α-related mitochondrial and Aβ effects, context-dependent for inflammatory pathways, and preliminary for tau phosphorylation, autophagic flux, and UPS-mediated clearance.

AD and PD have the deepest preclinical evidence; amyotrophic lateral sclerosis, Huntington’s disease, and MCI evidence is sparse, indirect, or matrix-based. Oral HT is rapidly absorbed but extensively conjugated, and in vitro BBB transport does not establish pharmacologically relevant human brain exposure.

Human isolated-HT trials demonstrate safety and selected systemic biomarker effects. Cognitive and BBB signals derive mainly from phenolic-rich olive oils, so they cannot establish isolated-HT efficacy. No adequately powered disease-specific trial has shown that isolated HT modifies AD, PD, or another neurodegenerative disorder.

Accordingly, available evidence is insufficient to recommend isolated HT for prevention or treatment of neurodegenerative disease. Standardized formulations, metabolite-resolved pharmacokinetics, direct human brain-exposure data, and disease-specific randomized trials with validated biomarkers and clinical outcomes are required.

## Figures and Tables

**Table 1:** Pharmacokinetic characteristics of HT after oral intake.

Pharmacokinetic Aspect	Main Observations	Evidence Type/Study Model	References
**Absorption and oral bioavailability**	HT is rapidly absorbed after oral intake, with plasma peaks typically occurring within 13–30 min. Exposure is influenced by the food matrix, particularly lipid-rich matrices such as extra virgin olive oil. Free HT concentrations are usually low because of rapid metabolism, and endogenous formation from secoiridoids may further contribute to systemic availability.	Human crossover pharmacokinetic studies	[10,39,43,45]
**Metabolism and circulating metabolites**	HT undergoes extensive phase II metabolism, mainly sulfation and glucuronidation. The main circulating and urinary derivatives include HT sulfates, HT glucuronides, HVA, HvOH, and DOPAC, with conjugated metabolites clearly predominating over the free compound.	Human pharmacokinetic and metabolite studies	[10,40,46,47]
**Half-life and systemic exposure**	Free HT shows extremely rapid plasma clearance after intravenous administration, whereas following oral intake, HT-derived metabolites remain detectable for several hours and may be excreted up to 12–24 h. The biological persistence of HT-related exposure may exceed the plasma half-life of the parent compound because circulating metabolites remain detectable beyond the disappearance of free HT; a contribution of metabolite recirculation has been proposed, although this remains less directly demonstrated in humans.	Animal i.v./oral and human oral studies	[10,47,48,49]
**Tissue distribution and brain exposure**	HT and its metabolites distribute rapidly to several organs, particularly liver and kidney, with lower but measurable levels detected in muscle, reproductive tissues and brain. Experimental evidence indicates that HT and some metabolites may reach the brain and accumulate in specific regions under certain conditions.	Rodent tissue-distribution studies	[50,52,53]
**Blood–brain barrier permeability**	Experimental studies using animal models and in vitro blood–brain barrier (BBB) systems indicate that HT can cross the BBB without disrupting barrier integrity. Brain exposure appears limited under physiological conditions but may increase when BBB permeability is altered or when delivery systems such as nanocarriers enhance transport efficiency.	Rodent and human in vitro BBB models	[8,53,54,56]
**Relevance of conjugated metabolites**	Conjugated metabolites represent the predominant circulating forms of HT and may contribute to biological activity through intrinsic antioxidant effects, systemic transport, and potential local deconjugation within target tissues.	Human metabolite and neuronal-cell studies	[8,10,57]

HT, hydroxytyrosol; HVA, homovanillic acid; HvOH, homovanillyl alcohol; DOPAC, 3,4-dihydroxyphenylacetic acid; BBB, blood–brain barrier.

**Table 2:** Molecular mechanisms underlying the anti-inflammatory activity of hydroxytyrosol and their pathophysiological relevance.

Mechanism	Molecular Targets	Experimental Model	Main Biological Effects	Pathophysiological Relevance	Ref.
Inhibition of innate immune signaling	TLR4, MyD88, NF-κB	DSS-induced colitis mice	Reduced inflammatory gene expression; restored epithelial barrier	Prevention of chronic intestinal inflammation	[73]
Modulation of MAPK signaling	ERK, JNK, p38 MAPK	Acute liver injury model	Decreased inflammatory mediator production	Limits inflammatory amplification	[74]
Suppression of pro-inflammatory cytokines	TNF-α, IL-1β, IL-6, IL-8, IL-17	In vitro/in vivo models	Reduced cytokine production	Attenuation of chronic inflammation	[6,73,74]
Inhibition of NLRP3 inflammasome	NLRP3, Caspase-1, Gasdermin D, IL-1β	DSS-induced colitis mice	Reduced pyroptosis and mucosal inflammation	Prevention of inflammation-driven tissue damage	[73]
Intestinal barrier protection	Tight junction proteins	Colitis models	Reduced permeability and endotoxin translocation	Gut homeostasis; prevention of systemic inflammation	[73]
Gut microbiota modulation	Beneficial/dysbiotic taxa	Animal models	Improved microbial composition	Regulation of gut–immune axis	[71,72,73,75]
Regulation of inflammaging	Autophagy pathways	Preclinical/review evidence	Reduced chronic low-grade inflammation	Mitigation of age-related inflammatory processes	[6]
Activation of antioxidant defenses	Nrf2, HO-1	Experimental models	Enhanced antioxidant response	Protection against oxidative-inflammatory disorders	[6]
Attenuation of neuroinflammation	Microglia, BBB	Aged mice; neurodegeneration models	Reduced neuroinflammation; enhanced neurogenesis	Prevention of cognitive decline	[6,70,72]
Clinical validation in humans	Antioxidant/anti-inflammatory status	Randomized controlled trial (overweight/prediabetes)	Improved antioxidant and anti-inflammatory status	Translational support for preclinical mechanisms	[12]

BBB, blood–brain barrier; DSS, dextran sulfate sodium; ERK, extracellular signal-regulated kinase; HO-1, heme oxygenase-1; IL, interleukin; JNK, c-Jun N-terminal kinase; MAPK, mitogen-activated protein kinase; MyD88, myeloid differentiation primary response protein 88; NF-κB, nuclear factor-kappa B; NLRP3, NOD-like receptor family pyrin domain-containing 3; Nrf2, nuclear factor erythroid 2-related factor 2; TLR4, Toll-like receptor 4; TNF-α, tumor necrosis factor-alpha.

**Table 3:** Comparative evidence for HT-related modulation of disease-associated protein aggregation.

Protein	HT-Specific Evidence	Representative Model	Evidence Strength	Ref.
Aβ	Interference with aggregation/toxicity; mixed effects on amyloid processing	In vitro Aβ1-42; TgCRND8 and APP/PS1 mice	Moderate preclinical	[100,111,112]
α-Synuclein	Remodeling/inhibition of assemblies; reduced toxicity	Biophysical/cellular assays; *C. elegans* and related tyrosols	Moderate; strongest direct protein evidence	[76,101,102,103]
Tau	Fibrillization and proteotoxicity signals; phosphorylation not established	In vitro olive derivatives; HT-rich *C. elegans* extract	Limited/preliminary	[63,106,113,114]

Aβ, amyloid-β; APP/PS1, amyloid precursor protein/presenilin 1; HT, hydroxytyrosol. Evidence strength reflects HT-specific support and does not imply human efficacy.

**Table 4:** Summary of preclinical evidence on hydroxytyrosol (HT) in models relevant to Parkinson’s disease.

Model/Approach	Compound Tested	Main Finding	Ref.
Microglial activation (in vitro)	HT	Reduced LPS- and α-synuclein-induced microglial activation	[76]
6-OHDA/dopamine toxicity, dopaminergic cells (in vitro)	HT	Nrf2-mediated phase II enzyme induction; cytoprotection	[128]
Salsolinol toxicity, PC12 cells (in vitro)	HT + synaptic vesicle proteins	Synergistic neuroprotection	[129]
BBB co-culture, hCMEC/D3–SH-SY5Y (in vitro)	HT (micellar nanocarriers)	Protection against oxidative stress; improved delivery	[56]
α-Synuclein aggregation, in vitro assay	HT, HTA, DOPAC	HTA fully blocked aggregation; outperformed HT and DOPAC	[102]
MPTP mouse model (in vivo)	HT	Restored striatal dopamine; improved motor performance	[130]
Rotenone mouse model (in vivo)	Hidrox^®^ (HT-rich extract)	Anti-inflammatory and antioxidant effects	[131]
MPP+ rat model (in vivo)	HT, HTA, nitro-HT	Attenuated oxidative damage (potency varied by derivative)	[132]
MPP+ rat model, MAO inhibition (in vivo)	HT	MAO isoform inhibition; prevented neurotoxicity	[133]
***C. elegans*** PD model, aggregation (in vivo)	HT, HTA	76.2% reduction in α-synuclein aggregation (HTA)	[102]
***C. elegans*** healthspan model (in vivo)	HT + oleuropein aglycone	Maintained healthspan; prevented PD-like phenotypes	[103]
Human BBB model, primary endothelial cells (in vitro)	HT	High permeability (65–100% transport in 24 h); sulfate metabolites formed	[55]
Narrative review, anti-inflammatory mechanisms	HT	Conceptual synthesis of anti-inflammatory action in PD	[7,134]
Non-PD model, inflammaging	HT	Modulation of autophagy and inflammatory signaling	[70]
Bioavailability review	HT	Factors and strategies affecting oral bioavailability	[10]

6-OHDA, 6-hydroxydopamine; BBB, blood-brain barrier; DOPAC, 3,4-dihydroxyphenylacetic acid; hCMEC/D3, human cerebral microvascular endothelial cell line D3; HT, hydroxytyrosol; HTA, hydroxytyrosol acetate; LPS, lipopolysaccharide; MAO, monoamine oxidase; MPP+, 1-methyl-4-phenylpyridinium; MPTP, 1-methyl-4-phenyl-1,2,3,6-tetrahydropyridine; nitro-HT, nitrohydroxytyrosol; Nrf2, nuclear factor erythroid 2-related factor 2; PC12, pheochromocytoma cell line; PD, Parkinson’s disease; SH-SY5Y, human neuroblastoma cell line.

**Table 5:** Quantitative comparison of representative experimental and human HT exposures.

Evidence Context	Representative Exposure	Model/End Point	Interpretation	Ref.
In vitro	1–50 μM HT	Neuronal/microglial and aggregation assays	Concentration; not a systemic dose	[76,128]
Rodent oral	2.95–5.85 mg/kg	Rat pharmacokinetics	Species- and route-specific exposure	[49]
Rodent i.v.	1.5 mg/kg	MPP+ rat; MAO activity	Non-oral; no human equivalence	[133]
Human chronic, isolated HT	5 or 15 mg/day; 15 mg/day	6 months; 16 weeks	Systemic/metabolic biomarkers; no neurological efficacy	[12,148]
Human acute	30.6 or 61.5 mg	PK and lipid-oxidation biomarkers	Single exposure; not chronic dosing	[40]

HT, hydroxytyrosol; i.v., intravenous; MAO, monoamine oxidase; MPP+, 1-methyl-4-phenylpyridinium; PK, pharmacokinetics. Using an HT molecular mass of 154.16 g/mol, 10–50 μM equals 1.54–7.71 mg/L, not mg/kg. Conversion to mg/kg requires distribution volume, route, absorption, and bioavailability and is therefore not valid from concentration alone.

## Data Availability

No new data were created or analyzed in this study. Data sharing is not applicable.

## Abbreviations

The following abbreviations are used in this manuscript:

6-OHDA	6-hydroxydopamine
8-OHdG	8-hydroxy-2′-deoxyguanosine
AD	Alzheimer’s disease
AMPK	AMP-activated protein kinase
ARE	antioxidant response element
Aβ	amyloid-β
BBB	blood–brain barrier
DOPAC	3,4-dihydroxyphenylacetic acid
DSS	dextran sulfate sodium
EFSA	European Food Safety Authority
HO-1	heme oxygenase-1
HT	hydroxytyrosol
HTA	hydroxytyrosol acetate
HVA	homovanillic acid
IL	interleukin
LDL	low-density lipoprotein
MAO	monoamine oxidase
MAO-B	monoamine oxidase B
MAPK	mitogen-activated protein kinase
MPP+	1-methyl-4-phenylpyridinium
MRI	magnetic resonance imaging
NADPH	nicotinamide adenine dinucleotide phosphate
NF-κB	nuclear factor-kappa B
NLRP3	NOD-like receptor family pyrin domain-containing 3
Nrf2	nuclear factor erythroid 2-related factor 2
PC12	pheochromocytoma cell line
PD	Parkinson’s disease
PGC-1α	peroxisome proliferator-activated receptor gamma coactivator 1-alpha
PI3K/Akt	phosphoinositide 3-kinase/protein kinase B
ROS	reactive oxygen species
SIRT1	sirtuin 1
SKN-1	skinhead-1 transcription factor
TLR4	Toll-like receptor 4
